# Weakly Supervised Building Semantic Segmentation Based on Spot-Seeds and Refinement Process

**DOI:** 10.3390/e24050741

**Published:** 2022-05-23

**Authors:** Khaled Moghalles, Heng-Chao Li, Abdulwahab Alazeb

**Affiliations:** 1 School of Information Science and Technology, Southwest Jiaotong University, Chengdu 610031, China; khaled.moghalles@gmail.com; 2 Department of Computer Science, College of Computer Science and Information Systems, Najran University, Najran 55461, Saudi Arabia; afalazeb@nu.edu.sa

**Keywords:** building semantic segmentation, deep learning, weakly supervised learning, very high resolution, imagery

## Abstract

Automatic building semantic segmentation is the most critical and relevant task in several geospatial applications. Methods based on convolutional neural networks (CNNs) are mainly used in current building segmentation. The requirement of huge pixel-level labels is a significant obstacle to achieve the semantic segmentation of building by CNNs. In this paper, we propose a novel weakly supervised framework for building segmentation, which generates high-quality pixel-level annotations and optimizes the segmentation network. A superpixel segmentation algorithm can predict a boundary map for training images. Then, Superpixels-CRF built on the superpixel regions is guided by spot seeds to propagate information from spot seeds to unlabeled regions, resulting in high-quality pixel-level annotations. Using these high-quality pixel-level annotations, we can train a more robust segmentation network and predict segmentation maps. To iteratively optimize the segmentation network, the predicted segmentation maps are refined, and the segmentation network are retrained. Comparative experiments demonstrate that the proposed segmentation framework achieves a marked improvement in the building’s segmentation quality while reducing human labeling efforts.

## 1. Introduction

Automatic building semantic segmentation in very high resolution (VHR) remote sensing images has proved use in a range of applications, including emergency management, urban planning, traffic evaluation, and mapping [1,2]. Segmentation is often used in computer vision [3,4] and industrial robots [5,6], but it has lately been used in remote sensing, which is important in a variety of applications such as environmental monitoring and danger identification [7]. Building segmentation using distant sensing photos (VHR images) is often more challenging than segmenting objects from ordinary photographs. Many factors, however, influence and complicate the extraction of 2D buildings from VHR photos, including sizes, backdrop complexity (i.e., water, shadow, vegetation, bodies, and other physical elements), roof diversities, and other topological difficulties [8]. For building extraction from two-dimensional and three-dimensional data, several techniques have been proposed, which include deep learning and traditional methods. In traditional methods, hand-crafted features, such as geometrical information and spectral/spatial information, are used [8,9]. In random field, clustering, and active contours, low-level features, such as color, texture, etc., are used [10,11]. However, they reduce representational ability and performance, and rely on an inefficient manual feature selection process.

Deep learning algorithms can extract high-level characteristics from 2D/3D data sets, harmonizing various absorption levels. As a result, deep learning dominates the field of building extraction [12,13]. A number of deep learning techniques have been developed for building extraction. The fully convolutional networks and the convolutional networks are often used as a foundation for newer image segmentation techniques [14]. Deeplab-V3 [15], VGG-16 [16], ResNet [17] and DensNet [18] are some of the pre-trained deep convolutional neural networks that have been designed to identify images.

Features are taken and integrated for each of the aforementioned networks to provide efficient segmentation. Furthermore, for the semantic segmentation of large things, abstract characteristics and high levels are utilized, whereas natural features and low levels are appropriate for tiny items. Several supervised semantic segmentation techniques based on deep networks have also been developed.

In semantic segmentation, the suggested approach for producing a building segmentation image assigns a class name to every pixel. To achieve outstanding results, deep neural networks must be trained with a high number of pixel-level segmentation labels. The most major constraint of the segmentation challenge is the collecting of pixel-level information. It will take some extra time and money because it is a bit challenging. Many researchers have developed a variety of DCNN-based weakly supervised segmentation approaches to lower the degree of pixel-level annotations. Only a few annotations, such as bounding boxes, image-level labels, and scribbles, are used in these techniques.

Although the image level label is the most time consuming and simple of all of these weakly supervised methods, the semantic segmentation accuracy is still considerably inferior to strongly supervised results when only image-level labels are used. Box-level annotations produce results that are quite analogous to real pixel-level annotations. However, box annotations include the object bounds and trusted background regions, and therefore box-supervised training is not possible for distributing information. Spot and scribble weakly supervised learning, on the other hand, occupies a center ground between image-level and box-level supervision. With spots, a few pixel locations are provided, which should lead to a higher level of performance than with image annotations [2,4,7,19]. A few extra pixels of location data provided by spots are expected to improve performance, compared to box-level annotations [18,20]. Spot seeds are more vague and lack a defined boundary of objects; compared to scribble [21], sparse spot seeds [21] are more efficient for annotating images. Additionally, spots are easier to note “things” (for example, sky, grass, ocean waters, and so on) that have hazy and ill-defined boundaries. In this paper, the training images are fed into a superpixels algorithm in order to forecast a boundary map.

**Figure 1:**
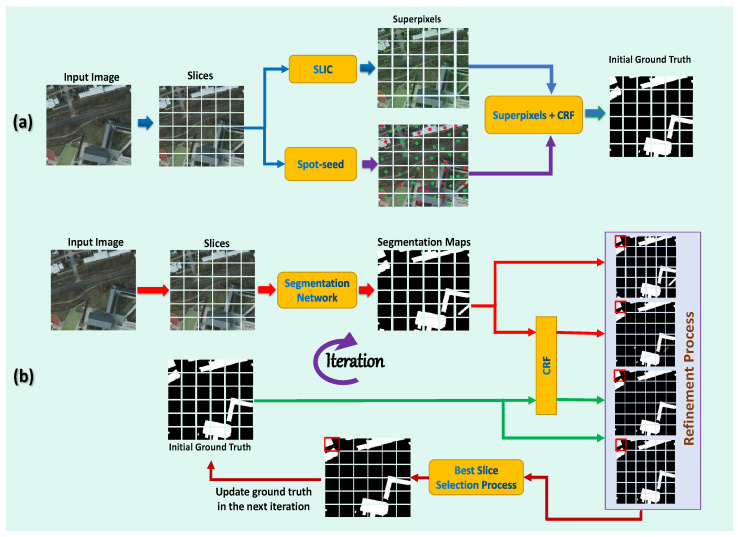
Pipeline of the proposed framework for the building’s semantic segmentation. (**a**) Generating initial ground truth: first, spot seeds are used to guide a superpixels-CRF model over superpixels segmentation to produce the initial ground truth. (**b**) Then, our framework utilizes the initial ground truth for the segmentation network training and predicts the segmentation masks of training images. In order to produce more accurate ground truth, we utilize a refinement process to smooth the segmentation network, which retrains again to provide more precise segmentation prediction as we iteratively optimize the segmentation.

The information from spot seeds is then propagated from spot seeds to unmarked regions, using a graphical model (superpixels-CRF) developed over superpixel regions to create the first pixel-level annotations, which can accommodate more boundaries and capture exact local structure while maintaining object shape. After that, the segmentation network is used to train and prophesy segmentation maps using the initial pixel-level annotations. The proposed refining technique is then used for segmentation masks in order to obtain precise and complete annotations at the pixel level, which are subsequently used to start training again the segmentation network. These steps are repeated continuously to provide high-quality annotations at the pixel level and to train a more accurate segmentation network. Our proposed method, as shown in Figure 1, enables more exact pixel-level annotations than earlier annotations, which improves the segmentation performance. The proposed method is known as the “spots supervised iteration framework (SSIF)” for weakly supervised building semantic segmentation in very high resolution (VHR images). Compared to previous fully supervised works, the proposed framework achieves comparable results while significantly reducing the annotation workload. To the best of our knowledge, this study is the first work to use spot annotations for weakly supervised building semantic segmentation. Our contributions to this work can be summarized as follows.

1. We release novel spot annotation datasets for building semantic segmentation.
2. We propose a method for generating high-quality pixel-level annotations using spot annotations and a graphical model based on superpixel segmentation.
3. A novel iterative training framework is proposed in our work. The performance can be improved by refining the pixel level annotation and iteratively optimizing the segmentation network.
4. According to experimental results on three public datasets, the proposed framework achieves a marked improvement in the building’s segmentation quality while reducing human labeling efforts.

The following chapters are organized as follows. Section 2 reviews related work in skin lesion segmentation. Section 3 elaborates on the mechanisms used in our framework. Section 4 demonstrates the experiment setting, results, analysis, etc. Finally, we conclude in Section 5.

## 2. Related Work

### 2.1. Semantic Segmentation of Remote-Sensing Images

Every pixel in an image is labeled using semantic segmentation techniques. In computer vision, semantic segmentation is used frequently. However, it has also recently become widely used in remote sensing. It has several applications, such as environment monitoring, natural hazard detection, urban planning, and land-cover classification [7]. Remote-sensing images have extremely high resolution and distinct properties compared to conventional digital images, which offer obstacles for semantic segmentation goals. Thus, semantic segmentation images require an efficient feature representation. The segmentation of remotely sensed images has been the subject of a considerable body of research. To create segments for images, traditional approaches, such as active contours and clustering, mean shifts, watersheds and Markov random field models, have been frequently employed. Hand-crafted feature selection is a frequent shortcoming of these systems that is difficult to optimize. Deep learning algorithms have lately demonstrated tremendous effectiveness on both remotely sensed and other images in semantic segmentation.

Semantic segmentation is presented using several deep learning architectures. SegNet [22,23] is a deep fully convolutional encoder–decoder architecture for semantic segmentation that has been proposed to be incorporated into a single framework. In SegNet, the lower connected layers of the network are replaced with convolutional layers, thus achieving robust end-to-end learning. Additionally, it employs an alternate decoder variant, which makes use of pooling indices to calculate the max-pooling step of the encoder through nonlinear sampling. As a result of this modification, the robustness of SegNet is improved, and sufficient memory is ensured. Another variant of the encoder–decoder model is U-Net [24,25], which enables the decoder to relearn key features that are lost during pooling in the encoder. A probabilistic graph model called conditional random field (CRF) was proposed in [26] to improve the output quality. CRF enhances the object boundary [27] and is used to further develop the network into an end-to-end trainable network. Semantic segmentation methods [5,6,28,29] have been helpful in the development of electrical monitoring systems for use in the electronic manufacturing industry.

### 2.2. Weakly Supervised Learning

Building segmentation has achieved excellent results in a fully supervised method in recent years, and segmentation performance has significantly increased. In order to reduce the time cost of data labeling and ensure the performance of the network, most recent studies for semantic segmentation focus on training models in semi-supervised or weakly supervised environments [30]. Image-level labels [31,32] or bounding boxes [33] have become the dominant weakly supervised setting. Jing et al. [31] proposed a new recursive coarse-to-fine semantic segmentation framework that requires only image-level annotations and allows for the generation of masks for multiple-labeled images, using a single class-labeled image. Dai et al. [33] leveraged bounding box annotations to iterate between automatically generating region proposals and training a convolutional network to gradually improve the performance of segmentation. In this work, we propose a weak supervision framework based on image-level labels that requires minimal labeling time but does not significantly impair performance.

## 3. The Proposed Method

**Figure 2:**
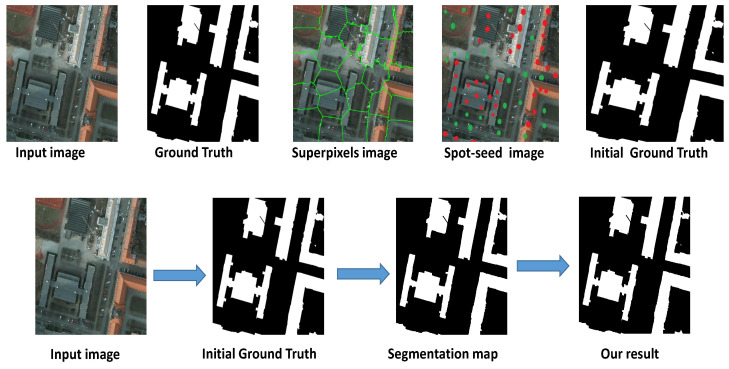
The main steps of the proposed framework.

This section introduces the training strategy for extracting buildings from VHR images using weakly supervised semantic segmentation, as well as the proposed framework in detail. The components of the proposed framework are first described. Second, we show how the initial ground truth annotations are made. Finally, we show how we may iteratively update the initial ground truth annotations using the refinement process and train the segmentation network. Figure 2 shows the main steps of the proposed method.

### 3.1. The Proposed Framework’s Architecture

A set of pixels with a category label is called an annotated spot seed with a category. The spot seeds are provided in a sparse manner, which is in contrast to the requirements of pixel-level semantic segmentation, which requires the identification of dense, internal, and integral regions in order to perform pixel-level inference. As a solution to this problem, we employ spot seeds to drive a superpixels-CRF model through superpixels segmentation, resulting in high-quality ground truths. Then, using the high-quality ground facts as supervision, the segmentation network is trained, and the anticipated segmentation masks are generated. The proposed refining approach is then applied to segmentation masks, resulting in more precise and full ground truths for retraining the segmentation network. These steps are repeated iteratively to obtain high-quality ground facts and improve the segmentation network.

### 3.2. Generating High-Quality Initial Ground Truths

The semantic segmentation criteria are not met because the spot seeds are too sparse, but they do offer position information for a few pixels of an object. With the aim of identifying the high-quality ground-truth value, a superpixels-CRF model is built over superpixels segmentation, which can propagate information from spot seeds to unmarked regions. We propose that these regions could potentially retain object contour, catch the deep local structure, and outperform spot seeds, which may include many little bits in the object segment but are not located on area bounds. We find that ground truth annotations obtained during the training stage with the proposed method will speed up network learning and provide more precise segmentation masks than spot seeds.

### 3.3. Spot-Seeds Guided Superpixels-CRF Model for Object Region Supplement

The superpixels-CRF model was utilized to disseminate data from spot seeds to unknown regions. To accomplish this, we create a network based on the superpixels segmentation. A vertex in the graph represents a region, and an edge in the graph represents the similarity between two regions. The proper segment image is denoted as *I*, and the $\left\{r_{k}\right\}$ is set of non-overlapping regions, which satisfies the condition $\cup_{k}\left(r_{k}\right)=I$ and $r_{k}\cap r_{m}=\phi ,\forall_{k,m}$. Moreover, spots of an input image are $C=\{c_{i},l_{i}\}$, where $c_{i}$ is the pixels of spot in category *i* and $0\leq l_{i}\leq L$ is the spot’s category label (supposing that there are *L* categories and $l_{i}=0$ for background). The region $r_{k}$ is used for a category label y∈{0,1,…,L}. Additionally, in order to determine the final label and minimize the energy, a graph-cut optimization framework [34] is used to find the final label, which minimizes the energy,

(1) $$E\left(label\left(I\right)\right)=\sum_{k}\psi_{u}^{spot}\left(label\left(r_{k}\right)\right)+\sum_{k,m}\psi_{p}\left(label\left(r_{k}\right),label\left(r_{m}\right)\right),$$

where $\psi_{u}^{spot}$ is a unary term that includes the region $r_{k}$ determined by the spot seed, and $\psi_{p}$ and is a pairwise term that connects two regions, $r_{k}$ and $r_{m}$. The following is the definition of the unary term:

(2) $$\psi_{u}^{spot}\left(label\left(r_{k}\right)=y\right)=\left\{\begin{array}{cc} 0, & \mathrm{if}\;y=l_{i}\;\mathrm{and}\;r_{k}\cap c_{i}\neq \phi \\ -log(\frac{1}{\left|\{l_{i}\}\right|}), & \mathrm{if}\;y\in \left\{l_{i}\right\}\;\mathrm{and}\;r_{k}\cap \left\{c_{i}\right\}=\phi \\ \infty , & \mathrm{otherwise} \end{array}\right.$$

According to the first condition in this equation, when a region $r_{k}$ overlaps with a spot seed $c_{i}$, the cost is zero when this region is allocated to the label $l_{i}$. On the contrary, when the region $r_{k}$ does not overlap with any spot having the same probability, $|\{l_{i}\}|\;$, denotes the number of spot labels on this image. This exclusive information is helpful in reducing false-positive predictions.

In this model, $\psi_{p}$, the pairwise term, indicates the similarity between two regions. Furthermore, it is seen as a simple look of similarity to its bordering regions. After that, we construct the histograms of the color and texter region $r_{k}$. The color histogram $h_{lab}\left(r_{k}\right)$ on area $r_{k}$ is based on the CIE Lab color space and is divided into 30 bins uniformly. The texture histogram $h_{t}\left(r_{k}\right)$ and a bank of 38 filters [35], including the Gaussian and Laplacian of Gaussian filters, edges and bar filters with three scales and six orientations, convolve the image. All bins are concatenated and standardized in color/texture histograms. If background pixels are near object spots and have a similar appearance to the object spots, or if background pixels are classified as object areas, object spots should be kept far away from them. This may have an impact on the segmentation quality. As a result, the pairwise term $\psi_{p}$ can be defined as follows:

(3) $$\begin{array}{c} \psi_{p}\left(label\left(r_{k}\right),label\left(r_{m}\right)\right)=\left[label\left(r_{k}\right)\neq label\left(r_{m}\right)\right].\\ exp\{-Simi\left(r_{k},r_{m}\right)\}, \end{array}$$

where [.] is 1 if the condition is met and 0 otherwise, and similarity is defined as

(4) $$Simi\left(r_{k},r_{m}\right)=Simi_{lab}\left(r_{k},r_{m}\right)+Simi_{t}\left(r_{k},r_{m}\right),$$

The color similarity and texture similarity are defined as

(5) $$Simi_{lab}\left(r_{k},r_{m}\right)=\frac{∥h_{lab}\left(r_{k}\right)-h_{lab}\left(r_{m}\right){∥}_{2}^{2}}{\lambda_{lab}^{2}},$$

(6) $$Simi_{t}\left(r_{k},r_{m}\right)=\frac{∥h_{t}\left(r_{k}\right)-h_{t}\left(r_{m}\right){∥}_{2}^{2}}{\lambda_{t}^{2}},$$

where $h_{lab}$ is the color histogram built on the CIE Lab color space, and $h_{t}$ is the texture histogram. In our experiment, we set empirically $\lambda_{lab}=5$ and $\lambda_{t}=10$. The definition implies that if the appearance of contiguous regions belonging to different labels is similar, the expenses will be higher. However, the labeling problem in Equation (1) is an NP-hard problem to solve. The expansion and swap moves technique [34], which determines the shortest cut for a given graphical model, can be used to solve it.

### 3.4. Network Training

To create segmentation masks, we use VGG16 [16] as our backbone network. As shown in Figure 1, we train the prediction network using initial ground truths. A discussion is held in Section 5 to explore the effectiveness of using VGG16 [16] compared to the other networks as the backbone. The cross-entropy loss is the loss function that promotes the prediction to match the real-world regions:

(7) $$\begin{array}{c} L_{s}\left(f_{l,c}\left(I|\theta \right),T,S_{c}\right)=-\frac{1}{\sum_{c=1}^{T}\left|S_{c}\right|}\sum_{c=1}^{T}\sum_{l\in S_{c}}log\left(f_{l,c}\left(I|\theta \right)\right), \end{array}$$

where $S_{c}$ is a collection of pixels in the supervision that are labeled with class *c*. To begin, we employ a VGG16-net [16] that is pre-trained on the ImageNet dataset [21]. Empirically, we select a learning rate of 0.0001 as our starting point. It takes an average of 50 epochs to converge. Stochastic gradient descent (SGD) with mini batch is used for the training classification and segmentation network. We set 0.5 as the dropout rate, 0.9 as the momentum, 0.0005 as the weight decay, and 12 as the batch size. After one iteration, we predict on the training dataset using the model with the lowest loss, and then refine the new predicted result using fully connected CRF [20]. The whole process iterates several times until the network finally converges. Our implementation is based on a NVIDIA GeForce TITAIN GPU with 12 GB memory.

### 3.5. The Proposed Refinement Process

Although the initial ground truth annotations are improved in accuracy, they are still distant from the true pixel-level annotations. The segmentation results obtained by training the segmentation network with initial ground truth annotations as supervision can be improved further. As a result, we introduce a refinement method in order to obtain more precise ground truth annotations. The original input image is denoted by the letter *I*, and the associated initial ground truth annotation is denoted by the letter $G_{anno}$. We use the trained model to generate segmentation maps after the initial complete training of the segmentation network is converged. We denote the predicted segmentation map as $S_{pred}$. In addition, we thoroughly couple the CRF operation to the initial ground truth annotations, as well as the projected segmentation maps. The segmentation maps that emerge are referred to as $C_{anno}$, and $C_{pred}$, respectively. According to Algorithm 1, we update the training samples as well as their related ground truth annotations for the following iteration. The CRF operation is denoted by CRF(), and $S_{update}$ signifies the updated ground truth annotation, which is then utilized as the segmentation ground truth for the next iterative training. The average pixelwise absolute difference between two segmentation maps (i.e., $S_{1}$ and $S_{2}$) is defined as APW(), which is determined as follows:

(8) $$\begin{array}{c} APW\left(S_{1},S_{2}\right)=\frac{1}{w\times h}\sum_{i=1}^{w}\sum_{j=1}^{h}\left|S_{1}\left(i,j\right)-S_{2}\left(i,j\right)\right|, \end{array}$$

the width and height of the segmentation map are *w* and *h*, respectively. We evaluate the mean APW() between each pair of initial ground truth annotations $G_{anno}$ after each training round. For the predicted segmentation map $S_{pred}$, when the mean APW() falls below a certain level or the total number of training rounds exceeds 5, the halting criteria are defined as the CRF output of the current segmentation map annotation $G_{anno}$, and the CRF output of the predicted segmentation map $C_{pred}$. We empirically set the thresholds, δ and θ to 15 and 40, respectively, during the annotation updating process, and we set the mean APW() for the training stop criteria at 0.05. The quality of segmentation maps is discussed in Section 5, with and without the proposed refinement process in order to demonstrate the influence of refined segmentation maps in terms of accuracy.


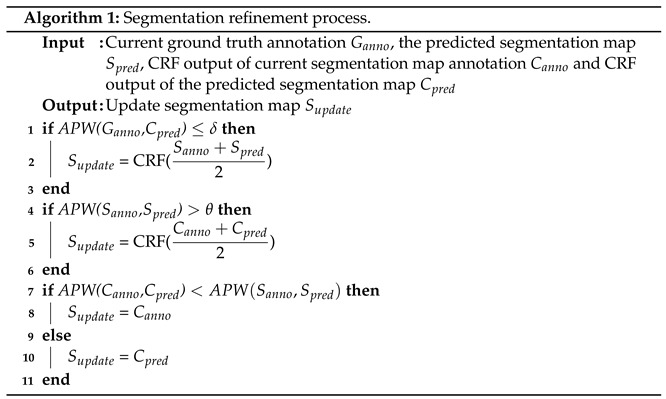


## 4. Experimental Results and Analysis

The effectiveness of the proposed method for building footprint segmentation is discussed in this section. The classification and segmentation network are trained and evaluated using Tensorflow on GPU (TITAIN). The goal of this framework is to bridge the gap between weakly and fully supervised semantic segmentation algorithms for building footprint segmentation. As a result, this gap remains an important measure of the effectiveness of weakly supervised semantic segmentation algorithms.

**ISPRS Potsdam Challenge Dataset (Potsdam) Dataset:** The ISPRS two-dimensional semantic label contest (Potsdam) is a standard dataset with accurate images, including 38 high-resolution actual orthophoto tiles chosen from a sizable TOP mosaic, which can be downloaded from the general website (https://www2.isprs.org/commissions/comm2/wg4/ (accessed on 7 May 2022 )). To increase the visibility of the small details, we adopt a tile that contains pixels size of 6000×6000 and a 5 cm resolution. The ground truth consists of 6 of the highest mutual land cover classes. For instance, buildings, invincible superficies, cars, plants, low vegetation, and clutter/background.

**WHU Building Dataset:** The WHU building dataset contains aerial and satellite subsets, as well as photos and labels for each, which can be downloaded from the general website (http://gpcv.whu.edu.cn/data/ (accessed on 7 May 2022)). For comparison with the proposed approach, we used an aerial subset that was widely used in previous studies. The data have 8189 images with 30 cm ground resolution and cover a $450\;{\mathrm{km}}^{2}$ area ${\mathrm{km}}^{2}$ in Christchurch, New Zealand. Each image is 512×512 and comprises three bands with pixels that correspond to red (R), green (G), and blue (B) wavelengths. The dataset broken into three sets: training (4736 images), validation (1036 images), and testing (2416 images). There are buildings, including 130,500, 14,500, and 42,000 tiles for the training, validation, and test datasets, respectively.

**Vaihingen Dataset:** The Vaihingen dataset is a public dataset for the ISPRS (2D) semantic labeling challenge dataset, which can be downloaded from the general website (http://www2.isprs.org/commissions/comm3/wg4/semantic-labeling.html/ (accessed on 7 May 2022)). The Vaihingen dataset includes 33 spectral orthoimages with annotated images. Each image has a resolution of 0.09 m and an average size of 2100×2100 pixels. These date were also chosen because the buildings have different shapes and sizes; the diversity of the elements that make up the roofs of the buildings; and also because there are similarities with the other components of the images.

### 4.1. Dataset Preprocessing

On the Potsdam and Vaihingen datasets, due to the limited GPU memory and the necessity for more samples in training, the images with the average size of (6000 × 6000) are divided into minimal patches of (256 × 256). Finally, we obtain training (18,122 images), validation (10,874 images), and testing (7249 images) for the Potsdam dataset, and training (4059 images), validation (2435 images), and testing (1624 images) for the Vaihingen dataset. We keep the original image size of 512×512 pixels in the WHU dataset and resize them to 256×256. Table 1 summarizes the characteristics of each dataset after preprocessing.

### 4.2. Evaluation

In this study, for the task evaluation, we employ pixel-based measures instead of object-based measures. The pixel-based technique works on the number of pixels in elicited buildings and determines the number of building while providing a quick and accurate estimate. The *F*1 score, lastly (MIOU) is used to measure the quantitative efficiency in the pixel-based evaluation. Hence, the *F*1 score can be computed as

(9) $$\begin{array}{c} F_{1}=2\times \frac{\mathrm{precision}\times \mathrm{recall}}{\mathrm{precision}+\mathrm{recall}}, \end{array}$$

where,

(10) $$\begin{array}{c} \mathrm{precision}=\frac{t_{p}}{t_{p}+f_{p}},\;\mathrm{recall}=\frac{t_{p}}{t_{p}+f_{n}}, \end{array}$$

where $t_{p}$, $f_{p}$, and $f_{n}$ are true positive, false positive, and false negative, respectively. These values can be calculated by the pixel-based confusion matrices per tile, or an accumulated confusion matrix. IoU is an average value of the intersection of the prediction and ground truth regions over their union, as follows. Then, the MIoU can be computed by averaging the *IoU* of all classes.

(11) $$\begin{array}{c} \mathrm{IoU}=\frac{\mathrm{precision}\times \mathrm{recall}}{\mathrm{precision}+\mathrm{recall}-\mathrm{precision}\times \mathrm{recall}}. \end{array}$$

### 4.3. Comparison with Other Methods on ISPRS Potsdam Challenge Dataset (Potsdam) Dataset

**Figure 3:**
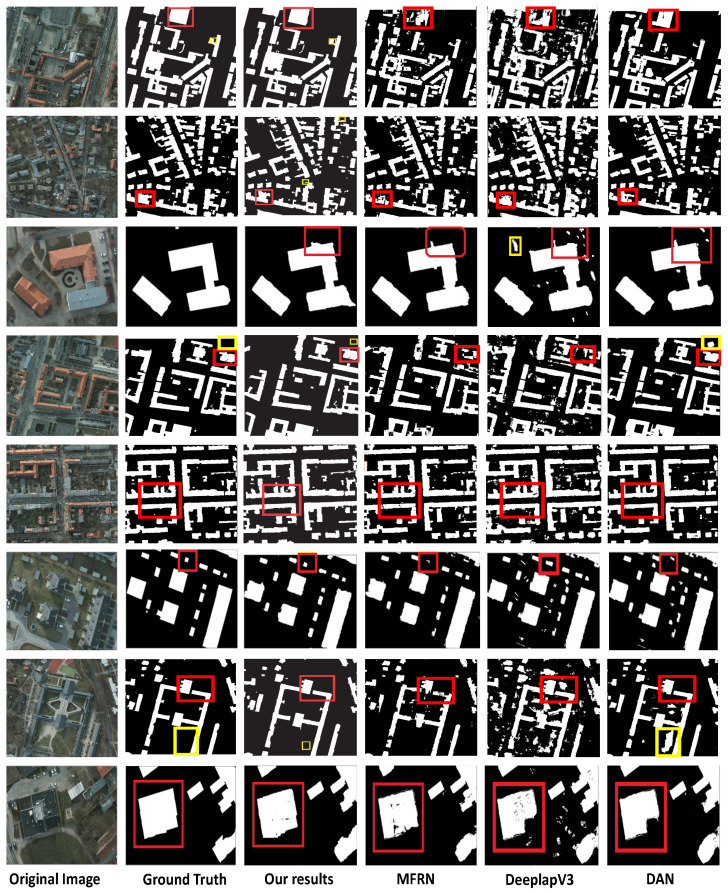
The building’s segmentation segmentation on Potsdam dataset. From left to right: original image, ground truth, our results, the multiple-feature reuse network (MFRN), Deeplab-V3, and the dense-attention network (DAN). The red boxes indicate improvement, while the yellow boxes indicate a false classification.

We compared the proposed weakly supervised method to other state-of-the-art fully supervised building footprint segmentation methods. The performance of building footprint segmentation is compared in Table 2, which shows that, while the proposed method’s various indicators are lower than other recently fully supervised and weakly supervised building footprint segmentation methods on the Potsdam dataset, the gap between the indicators is not big. Compared with these methods, the proposed method gives comparable results on most indications and greatly reduce the workload of annotation, demonstrating the effectiveness of the proposed method. Figure 3 shows the obtained results on the Potsdam dataset. The four approaches, as well as the Deeplab-V3 [15] MFRN [36] and DAN [14], are built and tested on the same empirical datasets (RGB images) used in the ISPRS 2D semantic-labeling contest (Potsdam). Nevertheless, several lower-level features of the Deeplab-V3 [15] and MFRN [36] networks have been overused, leading to over-segmentation due to limited spatial consideration; the fusion unit turns the produced fragmentary and minor buildings for five validity images. The boxes in red as indicated in Figure 3 exhibit the improvement gained after applying the proposed method. These results emphasize that the proposed method achieves comparable results. Moreover, the proposed method achieves remarkable performance in building extractions from the VHR images, despite a few false classified buildings (refer to the highlighted boxes in Figure 3.

### 4.4. Comparison with Other Methods on WHU Building Dataset

**Figure 4:**
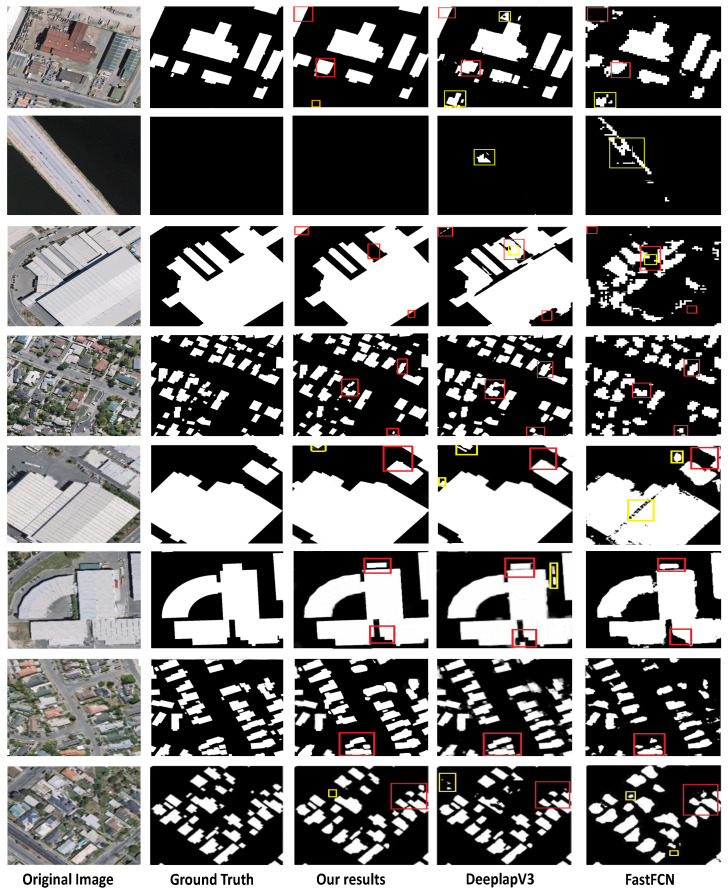
The building’s segmentation segmentation on WHU building dataset. From left to right: original image, ground truth, our results, Deeplab-V3, and FastCCN. The red boxes indicate improvement, while the yellow boxes indicate a false classification.

On WHU buildings dataset, we compare the obtained outcomes against FastFCN [39] and Deeplab-V3 [15] to describe the proposed method’s efficiency. The improvement obtained after using the proposed method is shown in red boxes in Figure 4. These findings demonstrate that the proposed method produces comparable outcomes also on the test images from the WHU dataset. The numerical performance indexes of several models are illustrated in Table 3. On all the four metrics, our proposed method produces comparable results compared to fully supervised and weakly supervised building footprint segmentation methods.

### 4.5. Comparison with Other Methods on Vaihingen Dataset

**Figure 5:**
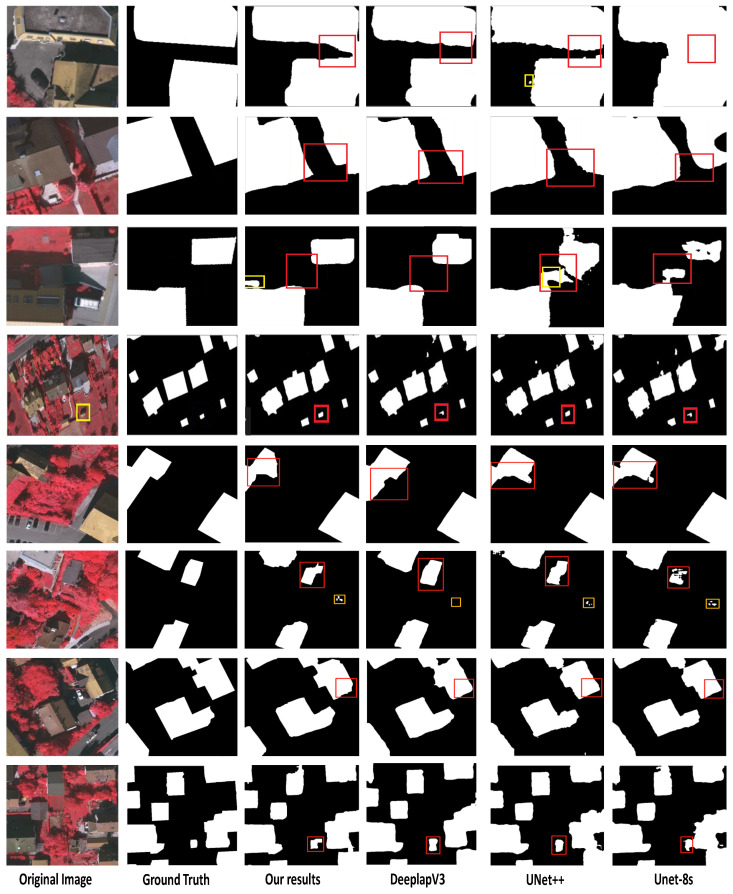
The building’s segmentation segmentation on Vaihingen dataset. From left to right: original image, ground truth, our results, Deeplab-V3, UNet++, and UNet-8s. The red boxes indicate improvement, while the yellow boxes indicate a false classification.

To investigate our model’s robustness and cross-dataset performance, we employ the Vaihingen dataset. As shown in Table 4, the proposed weakly supervised model performs well compared to the fully supervised methods, as shown in Figure 5. This demonstrates that the proposed framework has comparable accuracy and non-destructive segmentation ability, as well as good overall pixel-level segmentation performance. Furthermore, other methods are based on the concept of fully supervised learning and require a large number of manual annotation labels. The proposed weakly supervised framework not only reduces human efforts significantly, but it also outperforms some previous weakly supervised works in terms of some indicators.

## 5. Ablation Study

In this section, we explore the effectiveness of the proposed framework’s individual components.

### 5.1. The Influence of Backbone Networks

We conducted experiments using different backbone networks to evaluate its use. We found that by using Deeplab-V3 [15], it gives the best performance but it takes more time for training and inference, compared to using VGG16 [16], which gives the second best performance with less time, as shown in Table 5. For weakly supervised methods, the performance depends on the quality of ground truths. Therefore, it demonstrates the effectiveness of using VGG16 [16] in this study.

### 5.2. The Influence of Refinement Process

We conducted experiments with and without the refinement process to evaluate its effectiveness. This experiment involves training a segmentation network using initial ground truths, followed by the refinement process to refine the segmentation maps produced by the segmentation network. We find that with the refinement process, all metrics are improved, as shown in Table 6. It demonstrates that the proposed refinement process increases the accuracy of the initial ground truths and further enhances the performance of the segmentation network. The experiments demonstrate that the refinement process is useful to the segmentation task.

## 6. Conclusions

In this paper, we propose a new weakly supervised framework for building semantic segmentation. The framework first generates high-quality pixel-level labels, which are used as information to supervise the training of the network. In order to generate more precise pixel-level annotations, we use spot seeds to guide a graphical model construct over superpixel regions so that the information may be propagated to unmarked regions. These annotations at the pixel level are then used to supervise the network training and to forecast the segmentation performance. Compared to the initial annotations, the predicted result contains more complete regions of objects. The segmentation network is retrained using refined segmentation maps. The iterative training of these processes generates high-quality annotation information to be input into the subsequent segmentation network, making the training more accurate. The framework effectively reduces the gap between weakly supervised and fully supervised building semantic segmentation and reduces human labeling efforts. In future work, we will pay more attention to improving the quality of the initial annotations and developing weakly supervised approaches for building semantic segmentation.

## Figures and Tables

**Table 1:** Summary of the datasets used in this study.

Dataset Name	Total Images	Image Size	Train Set	Validation Set	Test Set
Potsdam	36,245	256×256	18,122	10,874	7249
WHU	8189	512×512	4736	1036	2416
Vaihingen	8118	256×256	4059	2435	1624

**Table 2:** The proposed network vs. other networks on Potsdam test set.

Methods	Recall (%)	Precision (%)	F1	IoU (%)
Deeplab-V3 [15] (fully)	88.89	83.00	83.36	79.37
MFRN [36] (fully)	86.24	74.43	91.80	89.74
DAN [14] (fully)	84.13	83.00	**92.56**	**90.56 **
Li et al. [37] (weakly)	**91.60**	87.60	89.50	81.00
ACGC [38] (weakly)	91.20	**92.00**	91.60	84.50
Ours (weakly)	84.05	77.15	87.45	85.65

**Table 3:** The proposed network vs. other networks on WHU building test set.

Methods	Recall (%)	Precision (%)	F1	IoU (%)
FastFCN [39] (fully)	81.37	87.98	84.55	73.23
Deeplab-V3 [15] (fully)	**92.99**	**93.11 **	**93.05**	**87.00**
Xin et al. (weakly) [40]	-	-	68.98	52.64
Ours (weakly)	86.75	87.02	85.45	82.34

**Table 4:** The proposed network vs. other networks on Vaihingen test set.

Methods	Recall (%)	Precision (%)	F1	IoU (%)
UNet [24] (fully)	90.66	91.95	94.98	91.58
UNet++ [41] (fully)	91.90	92.87	95.54	92.37
Deeplab-V3 [15] (fully)	**92.75**	**95.15 **	**96.73 **	**94.05**
Li et al. [37] (weakly)	84.50	83.60	84.10	72.50
ACGC [38] (weakly)	83.40	92.80	87.90	78.40
Ours (weakly)	88.02	90.89	91.75	89.34

**Table 5:** The computational cost of using different backbone networks on Potsdam dataset. Training and inference use images with a resolution of 256 × 256 pixels.

Backbone Network	MIoU (%)	Training Time/Image (s)	Inference Time/Image (s)
VGG16 [16]	75.24	**0.556**	**0.119**
Resnet-101 [42]	74.65	1.725	0.195
Deeplab-V3 [15]	**75.82**	2.986	1.563
UNet [24]	72.58	0.835	0.205

**Table 6:** The segmentation results with and without the refinement process.

Dataset	Training Type	Recall (%)	Precision (%)	F1	MIoU (%)
Potsdam	w/o the refinement process	65.34	69.28	71.42	63.47
	w/ the refinement process	**84.05**	**77.15**	**87.45**	**75.24**
WHU	w/o the refinement process	73.56	75.68	72.54	71.73
	w/ the refinement process	**86.75**	**87.02**	**85.45**	**82.34**
Vaihingen	w/o the refinement process	78.62	76.27	77.85	76.82
	w/ the refinement process	**88.02**	**90.89**	**91.75**	**89.34**
